# TNFα/TNFR2 signaling pathway: an active immune checkpoint for mesenchymal stem cell immunoregulatory function

**DOI:** 10.1186/s13287-020-01740-5

**Published:** 2020-07-16

**Authors:** Ghada Beldi, Maryam Khosravi, Mohamed Essameldin Abdelgawad, Benoît L. Salomon, Georges Uzan, Houda Haouas, Sina Naserian

**Affiliations:** 1 INSERM UMR-S-MD 1197, Hôpital Paul Brousse, Villejuif, France; 2 Paris-Saclay University, Villejuif, France; 3 National Institute of Applied Sciences and Technology (INSAT), Carthage University, LR18ES40, Inflammation, environment and signalization pathologies, Tunis, Tunisia; 4 Sorbonne Université, INSERM, CNRS, Centre d’Immunologie et des Maladies Infectieuses (CIMI-Paris), Paris, France; 5 Biochemistry Division, Chemistry department, Faculty of Science, Helwan University, Cairo, Egypt; 6 CellMedEx, Saint Maur Des Fossés, France

**Keywords:** Mesenchymal stem cells, Immune regulation, Tolerance induction, Regulatory T cells, TNFα/TNFR2 signaling pathway, Immune checkpoint

## Abstract

**Background:**

In addition to their multilineage potential, mesenchymal stem cells (MSCs) have a broad range of functions from tissue regeneration to immunomodulation. MSCs have the ability to modulate the immune response and change the progression of different inflammatory and autoimmune disorders. However, there are still many challenges to overcome before their widespread clinical administration including the mechanisms behind their immunoregulatory function. MSCs inhibit effector T cells and other immune cells, while inducing regulatory T cells (T regs), thus, reducing directly and indirectly the production of pro-inflammatory cytokines. TNF/TNFR signaling plays a dual role: while the interaction of TNFα with TNFR1 mediates pro-inflammatory effects and cell death, its interaction with TNFR2 mediates anti-inflammatory effects and cell survival. Many immunosuppressive cells like T regs, regulatory B cells (B regs), endothelial progenitor cells (EPCs), and myeloid-derived suppressor cells (MDSCs) express TNFR2, and this is directly related to their immunosuppression efficiency. In this article, we investigated the role of the TNFα/TNFR2 immune checkpoint signaling pathway in the immunomodulatory capacities of MSCs.

**Methods:**

Co-cultures of MSCs from wild-type (WT) and TNFR2 knocked-out (TNFR2 KO) mice with T cells (WT and TNFα KO) were performed under various experimental conditions.

**Results:**

We demonstrate that TNFR2 is a key regulatory molecule which is strongly involved in the immunomodulatory properties of MSCs. This includes their ability to suppress T cell proliferation, activation, and pro-inflammatory cytokine production, in addition to their capacity to induce active T regs.

**Conclusions:**

Our results reveal for the first time the importance of the TNFα/TNFR2 axis as an active immune checkpoint regulating MSC immunological functions.

## Background

MSCs represent heterogeneous population of non-hematopoietic [1], fibroblast-like multipotent stem cells [2–4]. Unlike many other stem cells that are tissue-specific, MSCs are present in various tissues, including bone marrow (BM), adipose tissue, amniotic fluid, endometrium, dental tissues, umbilical cord [2], and many others [5] in different species from mice to human [6].

MSCs are self-renewable, easily accessible, spindle-shaped cells that are expandable in vitro and show exceptional genomic stability [2, 7]. Their “stemness” is exemplified by their ability to differentiate, under certain physiological and experimental conditions [8], into multiple mesoderm cell types, including osteocytes, chondrocytes, adipocytes, and smooth muscle cells [9–11]. They can also differentiate into cell types of other germ layers, like neurons (ectoderm) and hepatocytes (endoderm) [9, 12, 13].

To identify MSCs in a mixed population of cells, the International Society for Cellular Therapy has proposed minimum criteria: adherence to plastic; expression of CD73, CD90, and CD105 markers; and the absence of the expression of CD14, CD34, CD45, HLA-DR monocyte, and hematopoietic markers [3, 6, 14, 15]. Moreover, they can express CD44 [1] and Sca1 (murine MSCs) markers [15]. Indeed, there are some interspecies differences regarding the expression of different mentioned markers. For example, CD105 and CD90 are strongly expressed on the majority of species including murine MSCs but are low on goat and sheep MSCs [16]. Finally, they must be able to differentiate in vitro into mesoderm cell types [17].

During the last decade, MSCs have drawn much attention for their application in regenerative medicine [18, 19]. This is mainly due to the fact that, among multipotent stem cells, uniquely MSCs can differentiate into different cell types from the three germ layers, to produce soluble growth factors and cytokines [20] and to maintain tissues homeostasis by replacing dead or dysfunctional cells [21]. Furthermore, some studies revealed that MSCs can stimulate resident cells and promote tissue regeneration [22].

Likewise, MSCs have generated considerable interest for exerting immunomodulatory functions. Several in vitro and in vivo studies indicated that MSCs strongly suppress immune cells in innate and adaptive immune systems [23, 24]. MSCs inhibit effector T cell proliferation and function in both autologous and allogenic conditions while activating and inducing T regs [25–27]. Consequently, they reduce pro-inflammatory cytokine production, including tumor necrosis factor alpha (TNFα) [8].

TNFα binds to two structurally distinct receptors, TNFR1 and TNFR2. TNFR1 is expressed ubiquitously, and its interaction with TNFα leads to apoptosis and cell death. Inversely, TNFR2 is expressed on limited cells, namely immune cells, endothelial cells, neural cells, and MSCs. Its interaction with TNFα leads to cell survival and proliferation [9, 28, 29]. Previous studies revealed that the TNFα/TNFR2 signaling in MSCs supports pro-angiogenic and protective mechanisms, and inversely, the TNFα/TNFR1 axis is involved in deleterious mechanisms. For example, murine TNFR2 KO bone marrow MSCs (BM-MSCs) showed less or no myocardial functional recovery in a rat model of acute ischemia accompanied by increased production of pro-inflammatory factors and reduced level of VEGF in the myocardium [30, 31]. This is consistent with other studies demonstrating that the production of VEGF, insulin-like growth factor 1 (IGF-1), and hepatocyte growth factor (HGF) by TNF-primed human BM-MSCs is TNFR2-dependent [32, 33]. Furthermore, the overexpression of the soluble form of TNFR2 (sTNFR2) or the membrane form of TNFR2 in human or rat BM-MSCs enhanced their therapeutic effects in murine models of RA [34, 35] and cardiac ischemia [36, 37], which was associated with reduced TNF, IL-1β, and IL-6 secretion.

Interestingly, many immunosuppressive cells like T regs, MDSCs, B regs, and EPCs express TNFR2 which is directly related to their immunosuppressive efficiency mostly through modulating the secretion of anti-inflammatory cytokines [38–41]. MSCs are susceptible to environmental changes, and their immunosuppressive function can be regulated when exposed to an inflammatory microenvironment [42]. The presence of TNFα and other pro-inflammatory cytokines prime MSCs towards more immunosuppressive functions [43–47].

To investigate the involvement of the TNFα/TNFR2 signaling pathway in the immunoregulatory effect of MSCs, we considered the following facts: (1) MSCs are among the rare population of TNFR2^+^ cells, and TNFα is important for their activation and immunomodulatory effect. (2) Many immunosuppressive cells express TNFR2 which is directly related to their immunosuppressive efficiency. Thus, we investigated if the expression of TNFR2 by MSCs is related to their proper immunoregulatory activities. We harvested BM-MSCs from TNFR2 KO mice and compared them with WT-MSCs expressing normal levels of TNFR2. Our results demonstrate for the first time that the TNFα/TNFR2 signaling pathway plays a critical role in the immunomodulatory effect of MSCs directly through higher suppression of T cells and indirectly via induction of more phenotypically active Foxp3^+^T regs.

## Methods

### MSC isolation, expansion, and characterization

BM-MSCs were isolated from the femurs and tibias of 6- to 8-week-old C57BL/6 WT mice (Charles River and Envigo) and C57BL/6 TNFR2 KO (B6.129S2-Tnfrsf1b^tm1Mwm^/J, The Jackson Laboratory). Mice were housed under pathogen-free conditions. Cells were cultured in 25-cm^2^ flasks in MEMα medium (Gibco) containing low glucose, 1% GlutaMAX, 10% FBS, and 1% penicillin/streptomycin/neomycin (P/S/N) (Gibco). Cells were incubated at 37 °C in a 5% CO_2_. Non-adherent cells were removed every 8 h; pure MSCs were obtained after 4–5 weeks. Cells were subcultured prior to confluency. In all experiments, WT and TNFR2 KO MSCs were used in passages 2 to 4.

For the identification of MSCs, 10^5^ cells/well WT and TNFR2-MSCs were seeded in Falcon 96-well round-bottom plates. They were then immunostained with CD44-PeCy7, Sca1-APC, CD105-FITC, CD73-PE, CD45-VIOBLUE, CD34-Biotin or FITC, CD90-Biotin or PE and anti-biotin-PE or VIOBLUE (Miltenyi), and streptavidin-PeCY5/CY7 (eBioscience). Unstained cells and isotypes were used as controls. Flow cytometric analysis was performed using the LSRFORTESSA flow cytometer (BD-Biosciences) and analyzed using the FlowJo software v10 (FlowJo-LLC).

### MSC differentiation assay

WT and TNFR2 KO-MSCs were tested for their ability to differentiate into adipocytes and osteoblasts. Adipogenic differentiation was induced by culturing cells in a specific differentiation homemade medium for 21 days as already described [48]. Cells were then stained with Oil Red for 5 min. For osteocyte differentiation, MSCs were cultured in differentiation medium (StemXVIVO Osteogenic/Adipogenic Base Media supplemented with P/S/N at 1:100 dilution + StemXVIVO Mouse/Rat Osteogenic Supplement 20×) (R&D Systems) for 17 days and stained 3 min with 2% Alizarin Red.

### T cell isolation and culture

Pan T cell isolation kit  II (Miltenyi) was used to isolate total CD3^+^T cells from pooled spleens and lymph nodes of 6- to 12-weeks-old female WT C57BL/6 mice (Envigo and Charles River) and TNFα KO mice (B6.129S-Tnf^tm1Gkl^/J, The Jackson Laboratory). CD25^+^ cells were depleted from the CD3^+^T cell population using an anti-CD25 biotin-conjugated antibody (7D4, BD-biosciences), followed by staining with anti-biotin microbeads (Miltenyi). Subsequently, cells were isolated using magnetic-activated cell sorting (MACS). The resulting CD3^+^CD25^−^T cells, ≥ 92% pure, were cultured in the presence of WT and TNFR2 KO-MSCs.

### MSC/T cell co-culture

WT or TNFR2 KO-MSCs were seeded into 6-well or 12-well plates and incubated for 24 h in MEMα containing low glucose, 1% GlutaMAX, 10% FBS, and 1% P/S/N (Gibco). CD3^+^CD25^−^T cells were then added in different ratios, depending on experimental conditions, in RPMI medium containing 10% FBS, 1% P/S/N, 1% HEPES, and 0.1% β-mercaptoethanol. All co-cultures were performed in 50% MEMα-50% RMPI media. T cells were collected after 3 days by gentle aspiration. The isolation of T cells from co-culture is based on MSCs’ ability to adhere to plastic; however, T cells stay in suspension.

### T lymphocyte proliferation assay

5 × 10^4^ WT or TNFR2 KO-MSCs were co-cultured in 6-well plates with increasing numbers of mice WT-CD3^+^CD25^−^T cells (responder cells) in a total volume of 3 ml. The ratios of MSCs to T cells were 1/1, 1/2, 1/4, 1/6, 1/8, and 1/10. 2 × 10^5^ WT CD3^+^CD25^−^T cells were used as control T cells alone. Cells were labeled with carboxy fluorescein succinimidyl ester (CFSE) (Thermo Fisher) and stimulated by Dynabeads Mouse T-Activator CD3/CD28 (Gibco) according to the supplier’s protocol. After 3 days, T cells were collected and immunostained with CD4-VIOBLUE and CD8α-PeCy7 (Miltenyi). To eliminate the possibility of a non-specific effect of media on T cell proliferation, T cells were cultured in 50% RPMI-50% MEMα and compared to growth in 100% RPMI.

### T lymphocytes activation quantification

3 × 10^4^ WT or TNFR2 KO-MSCs were co-cultured in 12-well plates with 1.5 × 10^5^ (1/5 ratio) of mice WT-CD3^+^CD25^−^T cells in a total volume of 2 ml. We used this intermediate fixed ratio since we observed 50% immunosuppressive activity by MSCs. WT-CD3^+^CD25^−^T cells were stimulated by Dynabeads Mouse T-Activator CD3/CD28 (Gibco). 2 × 10^5^ freshly isolated WT-CD3^+^CD25^−^T cells were used as control T cells alone. After 3 days, WT-CD3^+^CD25^−^T cells were harvested and immunostained with CD4-VIOBLUE and CD8α-PeCy5 or CD8α-FITC, ICOS-PeCy7, GITR-PE, CD25-PeCy7 or PE, and TNFR2-APC (Miltenyi). Intracellular Foxp3 staining was performed according to the manufacturer’s instructions, using the Foxp3 staining buffer set kit (eBioscience).

### T lymphocyte cytokine quantification

3 × 10^4^ WT or TNFR2 KO-MSCs were co-cultured in 12-well plates with 1.5 × 10^5^ (1/5 ratio) of fresh mice WT CD3^+^CD25^−^T cells in a total volume of 2 ml. After 3 days, WT-CD3^+^CD25^−^T cells were harvested. Cells were then stimulated with 1 μg/ml PMA and 0.5 μg/ml ionomycin for 4 h and 30 min (Sigma), in the presence of 1 μl/ml GolgiPlug for the last hour (BD Biosciences). They were then immunostained with CD4-VIOBLUE, CD8α- Pe-Cy7, IFNγ-APC, TNFα-FITC, IL-10-APC, IL-17-PE, IL-2-FITC (Miltenyi), and anti-TGFβ-PE (Biolegend).

### T reg induction assay

3 × 10^4^ WT or TNFR2 KO-MSCs were co-cultured in 12-well plates with increasing numbers of mice WT-CD3^+^CD25^−^T cells in a total volume of 2 ml. The ratios of MSCs to T cells were 1/1, 1/2, 1/4, 1/6, 1/8, and 1/10. 2 × 10^5^ WT-CD3^+^CD25^−^T cells were used as control T cells alone. T cells were stimulated with Dynabeads Mouse T-Activator CD3/CD28 (Gibco). For the condition using T cells from TNFα KO mice, 3 × 10^5^ TNFα KO CD3^+^CD25^−^T cells were activated with Dynabeads Mouse T-Activator CD3/CD28 and co-cultured with 3 × 10^4^ WT-MSCs (1/10 ratio MSCs/T cells). After 3 days, T cells were collected and immunostained using the following Abs: CD4-VIOBLUE, CD8α-FITC, CD25-PE-Cy7, CTLA4–PE, TNFR2-APC (Miltenyi), and Foxp3-PE-Cy5 (eBioscience).

### Statistical analysis

Prism (GraphPad) was used for statistical analysis. Shapiro-Wilk normality test was performed to assess the normal distribution of data. Then, Student *t* test or 1-way ANOVA with post hoc analysis was performed depending on the number of comparatives. For cytometry analysis, we have normalized the MFI values with T cell alone control group. Then, we used unpaired, 2-tailed Student *t* tests or 1-way ANOVA for *P* value generation.

## Results

### MSC characterization

**Fig. 1:**
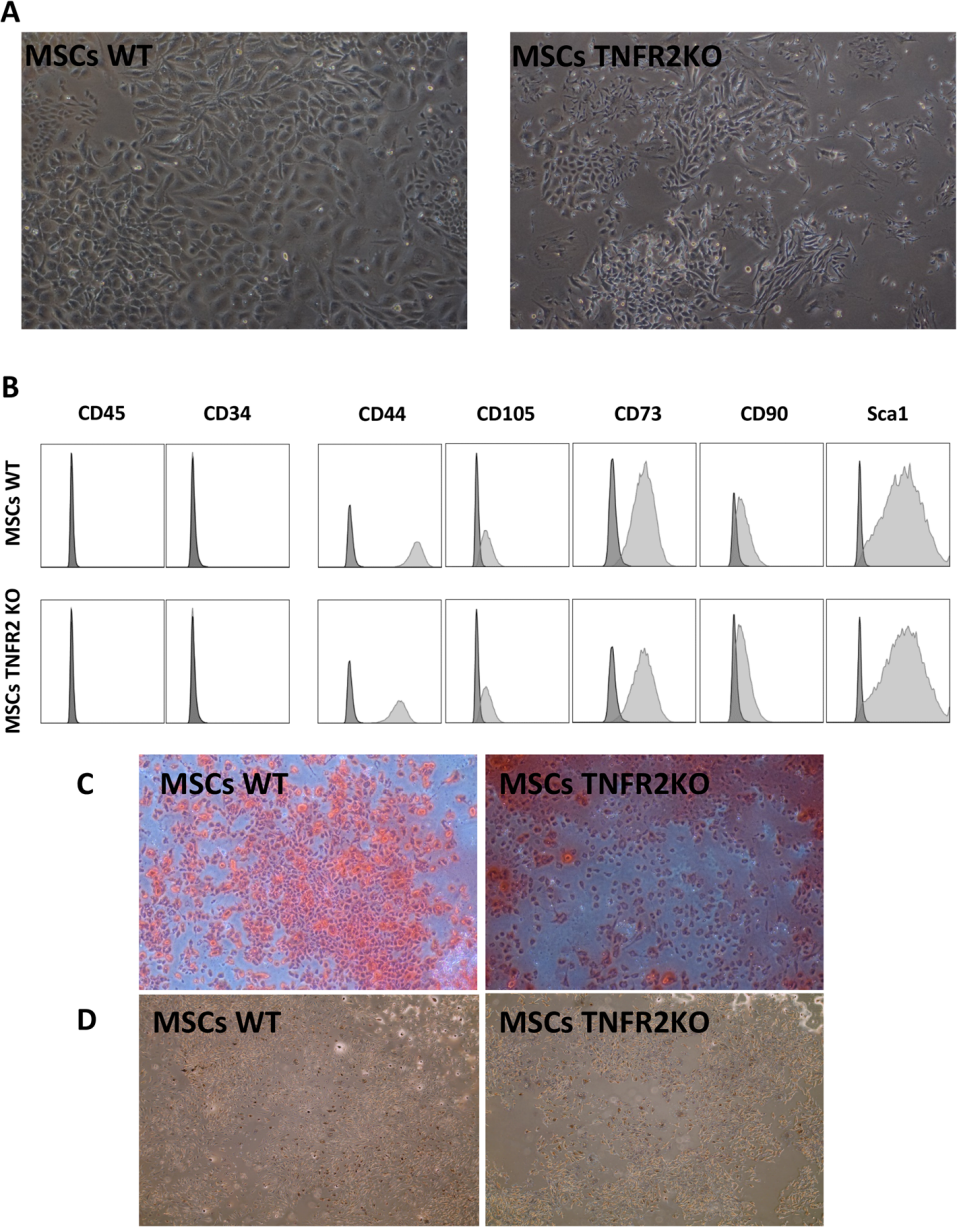
MSC WT and TNFR2 KO characterization. **a** MSCs WT showed normal spindle-shaped fibroblast-like appearance (passage 3) (× 4) while MSCs TNFR2 KO exhibited a more heterogeneous morphology (passage 3) (× 4). **b** Flow cytometry analyses of the surface expression of CD45, CD34, CD44, CD105, CD73, CD90, and SCA1 in MSCs WT and TNFR2 KO (passage 3). Both MSC populations were negative for CD45 and CD34 and positive for the rest of the markers studied. The dark gray histograms represent isotype controls. Data are representative of *n* = 6 in 3 independent experiments. **c** Osteogenic differentiation: both MSCs WT and TNFR KO (passage 3) were incubated in osteogenic differentiation medium for 17 days followed by Alizarin Red S staining (× 4). **d** Adipogenic differentiation: both MSCs WT and TNFR2 KO (passage 3) were incubated in adipogenic differentiation medium for 21 days followed by Oil Red O staining (× 4)

First, we assessed if BM-MSCs harvested from WT and TNFR2 KO mice are pure cells with normal physiological functions. Both were able to adhere to plastic plates and proliferate until late passages. While WT-MSCs showed normal morphological appearance, TNFR2 KO-MSCs were more heterogeneous with lower proliferation rate at passages 0 and 1 (Fig. 1a). The proliferation rate became equivalent to that of WT-MSCs in latter passages (data not shown). Moreover, both WT and TNFR2 KO-MSCs were positive for murine MSC markers such as CD44, CD105, CD73, CD90, and Sca-1 and negative for CD34 and CD45 markers (Fig. 1b). In addition, we demonstrated their capacity to differentiate into osteocytes and adipocytes under appropriate conditions (Fig. 1c, d).

### The MSC suppressive effect is decreased in the absence of the TNFα/TNFR2 signaling pathway

**Fig. 2:**
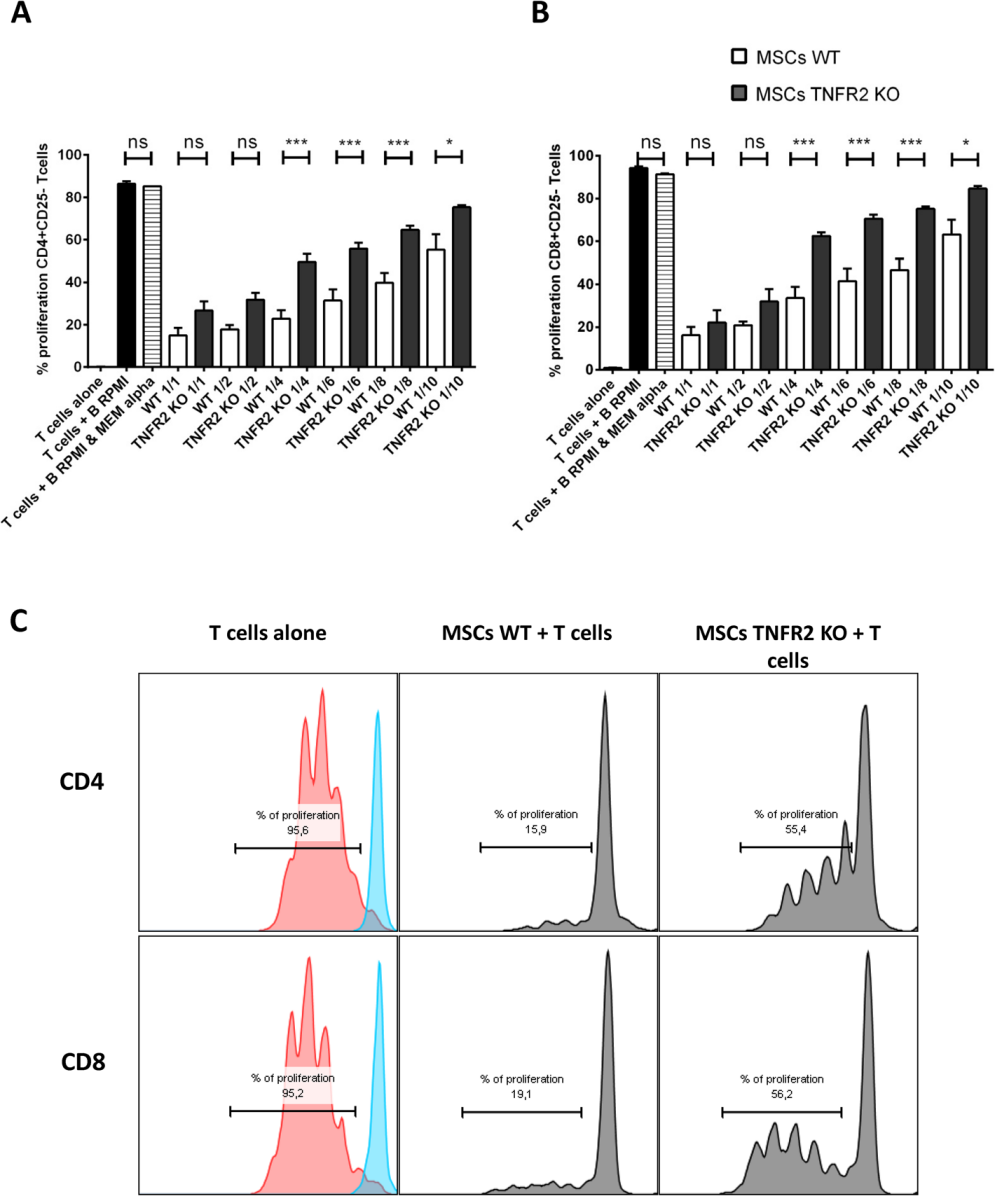
MSCs’ suppressive effect is diminished in the absence of the TNFα/TNFR2 signaling pathway. Activated CFSE^+^ CD4^+^ or CD8^+^ effector T cells were co-cultured with MSCs WT or TNFR2 KO in different MSC/T cell ratios (*n* = 6). Proliferation of CD4^+^ T cells (**a**) and CD8^+^ T cells (**b**) was measured by flow cytometry. The first bar represents the unstimulated T cells alone (*n* = 6), the second bar represents the bead-stimulated T cells alone in RPMI (*n* = 8), and the third bar is for the stimulated T cells alone in 50% RPMI+50% MEMα (*n* = 6). All data are collected from 3 different experiments. **c** A flow cytometry representative of proliferation assay at a 1:4 MSC/T cell ratio. Non-stimulated T cells have a single peak represented in blue. Upon stimulation, T cells alone or in co-culture with MSCs start to proliferate, and one can see less intensity of CFSE fluorescence. Data are represented as mean value ± SEM. One-way ANOVA analysis was performed to generate *P* values. ns, non-significant; *P < .05, **P < .01, ***P < .001, ****P < .0001. **b** Anti-CD3 and anti-CD28 activation beads

MSCs inhibit T cell proliferation in response to mitogenic stimuli and CD3/CD28 stimulation. Here, we investigated the role of the TNFα/TNFR2 axis in the ability of MSCs to suppress T cell proliferation. WT and TNFR2 KO-MSCs were co-cultured with CFSE^+^CD3^+^CD25^−^T cells in 6 different ratios. CD25^+^T cells were depleted from the primary population of T cells to eliminate activated T cells and non-specific immunosuppression by T regs. After 3 days, T cells were collected and the proliferation capacity of CD4^+^ and CD8^+^populations was quantified. Since two different culture media were used for T cells and MSCs (RPMI and MEMα, respectively), we used a 1:1 mix in co-culture conditions. No difference was observed between T cells cultured in 100% RPMI or in 50% MEMα-50% RPMI (Fig. 2a, b). In all the ratios, CD4^+^CD25^−^ (Fig. 2a) and CD8^+^CD25^−^ (Fig. 2b) responder T cells in co-culture with WT or TNFR2 KO-MSCs proliferated less than anti-CD3/CD28-activated T cells alone (T cells + B RPMI). Interestingly, we observed that WT-MSCs were significantly more immunosuppressive than TNFR2 KO-MSCs for both CD4 and CD8 T cell proliferation (Fig. 2a–c). A significant difference in immunosuppressive effect between WT and TNFR2 KO-MSCs was observed starting from 1/4 ratio for CD4^+^T cells (22.91% and 49.60% of proliferation, respectively) and also for CD8^+^ T cells (33.56% and 62.41% of proliferation, respectively). This difference was obvious until 1/10 ratio for CD4^+^T cells (55.28% and 75.31% of proliferation, respectively) and for CD8^+^T cells (63.13% and 84.63% of proliferation, respectively). This is the first evidence of a significant dose-dependent immunosuppressive effect of MSCs on T cells that is more accentuated in WT-MSCs than in TNFR2 KO-MSCs. However, the absence of TNFR2 did not entirely abolish the MSC immunosuppressive potential.

### Expression of TNFR2 on MSCs is correlated to increased CD4^+^ conventional T cell immunomodulation

**Fig. 3:**
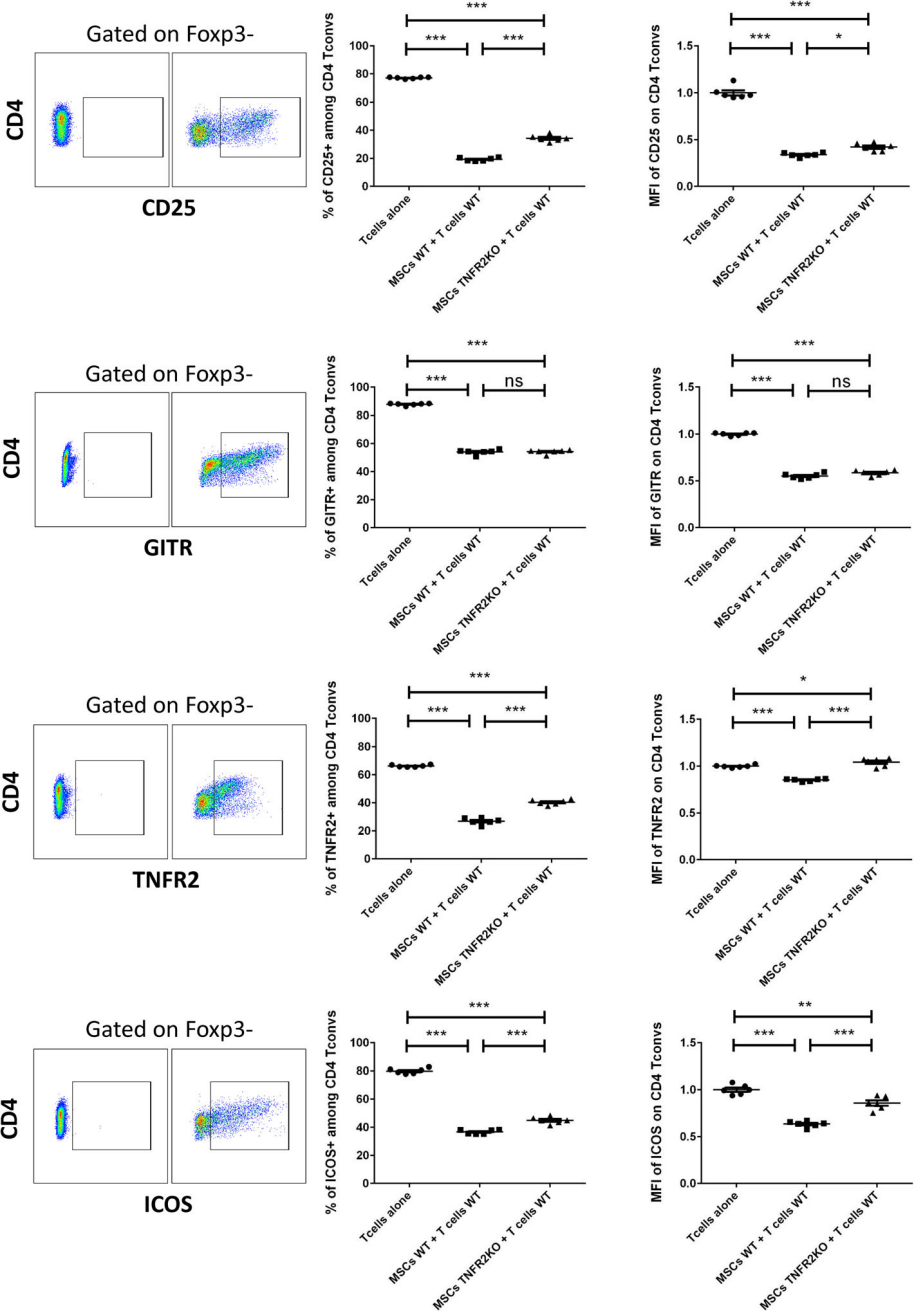
The expression of TNFR2 on MSCs is directly correlated to their higher immunomodulation of CD4^+^ T convs. Activated CD3^+^ effector T cells were co-cultured with MSCs WT or TNFR2 KO in a fixed 1/5 ratio. After 3 days, T cells were collected and different activation markers were studied. Cells were gated on CD4^+^Foxp3^−^ T conventional cells. For each marker, the strategy of gating is indicated on the left (*y*-axis) and below (*x*-axis) the figure. The *x*-axis represents each activation marker, and the *y*-axis represents the CD4 population. Left dot plots represent the Fluorescence Minus One (FMO) controls, and right dot plots represent the main samples. Each dot represents a measured value (*n* = 6) collected from 2 different experiments. For each group of values, horizontal lines represent the mean value and standard error of the mean. MFI values have been normalized with the T cells alone control group. One-way ANOVA analysis was performed to generate *P* values. ns, non-significant; *P < .05, **P < .01, ***P < .001, ****P < .0001

We next investigated if the ability of MSCs to modulate the activation profile of CD4^+^T cells is also TNFR2 dependent. WT and TNFR2 KO-MSCs were co-cultured for 3 days with anti-CD3/CD28-activated WT-CD3^+^CD25^−^T cells at a fixed 1/5 ratio (MSCs/T cells). T cells were then collected and analyzed for the percentage of expression and the mean fluorescence intensity (MFI) of different activation markers for conventional CD4^+^Foxp3^−^T cells (CD4 T convs). We first measured the expression of CD25; the α-chain of the IL-2 receptor constitutively expressed on the surface of T reg and activated T convs [46, 48]. After 3 days, we observed a dramatic decrease in the percentage of CD25^+^ cells and CD25 expression level in CD4^+^T convs when co-cultured with WT or TNFR2 KO-MSCs (Fig. 3). However, this decrease was significantly higher for WT than TNFR2 KO-MSCs. We also evaluated the expression of two members of the TNFα receptor superfamily, GITR (TNFRSF18) and TNFR2 (TNFRSF1B), both important in T cell biology and expressed by activated T cells [49, 50]. Three days after, we observed a decrease in the percentage of GITR^+^ cells and MFI of GITR on CD4^+^T convs compared to T cells alone. No significant difference was observed between WT and TNFR2 KO-MSCs (Fig. 3). For TNFR2 expression, we noticed a significant decrease in the percentage of TNFR2^+^ cells among CD4^+^T convs when co-cultured with WT or TNFR2 KO-MSCs compared to T cells alone (Fig. 3). This decrease was more important for WT than for TNFR2 KO-MSCs. However, for MFI of TNFR2 on CD4^+^T convs, we noticed a decrease when co-cultured with WT but an increase with TNFR2 KO-MSCs. Finally, we studied the expression of ICOS among CD4^+^T convs. ICOS co-stimulatory receptor is essential for T cell activation and proliferation [51]. After 3 days of co-culture, we observed a significant reduction in the percentage of ICOS^+^ cells and ICOS expression level among CD4^+^T convs when co-cultured with WT or TNFR2 KO-MSCs (Fig. 3). This decrease was significantly more important for T cells co-cultured with WT than with TNFR2 KO-MSCs.

### Expression of TNFR2 on MSCs is correlated to increased CD8^+^ conventional T cell immunomodulation

**Fig. 4:**
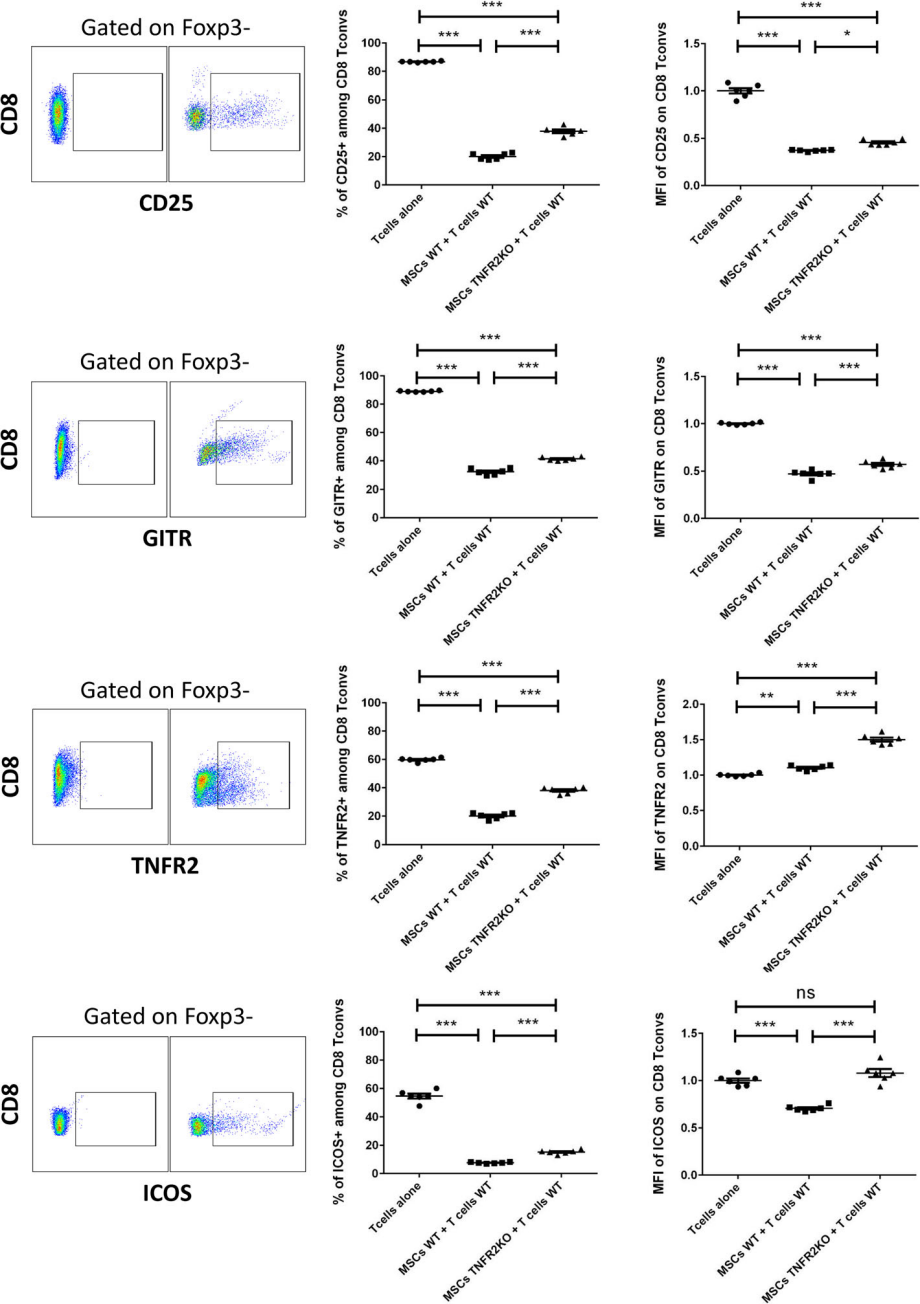
The expression of TNFR2 on MSCs is directly correlated to their higher immunomodulation of CD8^+^ T convs. Activated CD3^+^ effector T cells were co-cultured with MSCs WT or TNFR2 KO in a fixed 1/5 ratio. After 3 days, T cells were collected and different activation markers were studied. Cells were gated on CD8^+^Foxp3^−^ T conventional cells. For each marker, the strategy of gating is indicated on the left (*y*-axis) and below (*x*-axis) the figure. The *x*-axis represents each activation marker, and the *y*-axis represents the CD8 population. Left dot plots represent the Fluorescence Minus One (FMO) controls, and right dot plots represent the main samples. Each dot represents a measured value (*n* = 6) collected from 2 different experiments. For each group of values, horizontal lines represent the mean value and standard error of the mean. MFI values have been normalized with the T cells alone control group. One-way ANOVA analysis was performed to generate *P* values. ns, non-significant; *P < .05, **P < .01, ***P < .001, ****P < .0001

To investigate the role of TNFR2 expression by MSCs on the cytotoxic T cell activation profile, WT and TNFR2 KO-MSCs were co-cultured for 3 days with anti-CD3/CD28 activated WT-CD3^+^CD25^−^T cells at a 1/5 ratio (MSCs/T cells). T cells were then collected and analyzed for the percentage of expression and MFI of different activation markers of conventional CD8^+^Foxp3^−^T cells (CD8 T convs). We observed a dramatic decrease in the percentage of CD25^+^ cells and CD25 expression level in CD8^+^T convs when co-cultured with WT or TNFR2 KO-MSCs (Fig. 4). However, this effect was more accentuated in the presence of WT-MSCs versus TNFR2 KO-MSCs. Furthermore, analysis of GITR showed a decrease in the percentage of GITR^+^ cells and MFI of GITR on CD8^+^T convs. This decrease was significantly more important for WT than TNFR2 KO-MSCs. For TNFR2, we observed a decrease in the percentage of TNFR2^+^ cells in CD8^+^T convs co-cultured with WT or TNFR2 KO-MSCs (Fig. 4). Once again, this decrease was more significant for WT-MSCs versus TNFR2 KO-MSCs. We found no decrease in MFI of TNFR2 but a significant increase when co-cultured with TNFR2 KO-MSCs. Finally, we studied the expression of ICOS on CD8^+^T conv. There was a notable decrease in the percentage of ICOS^+^ cells, when co-cultured with WT or TNFR2 KO-MSCs. This decrease was more important with WT-MSCs than TNFR2 KO-MSCs. A significant decrease in MFI of ICOS on CD8^+^T convs was observed only when co-cultured with WT-MSCs (Fig. 4).

### MSCs modulate the capacity of T cells to produce pro- and anti-inflammatory cytokines via the TNFα/TNFR2 signaling pathway

**Fig. 5:**
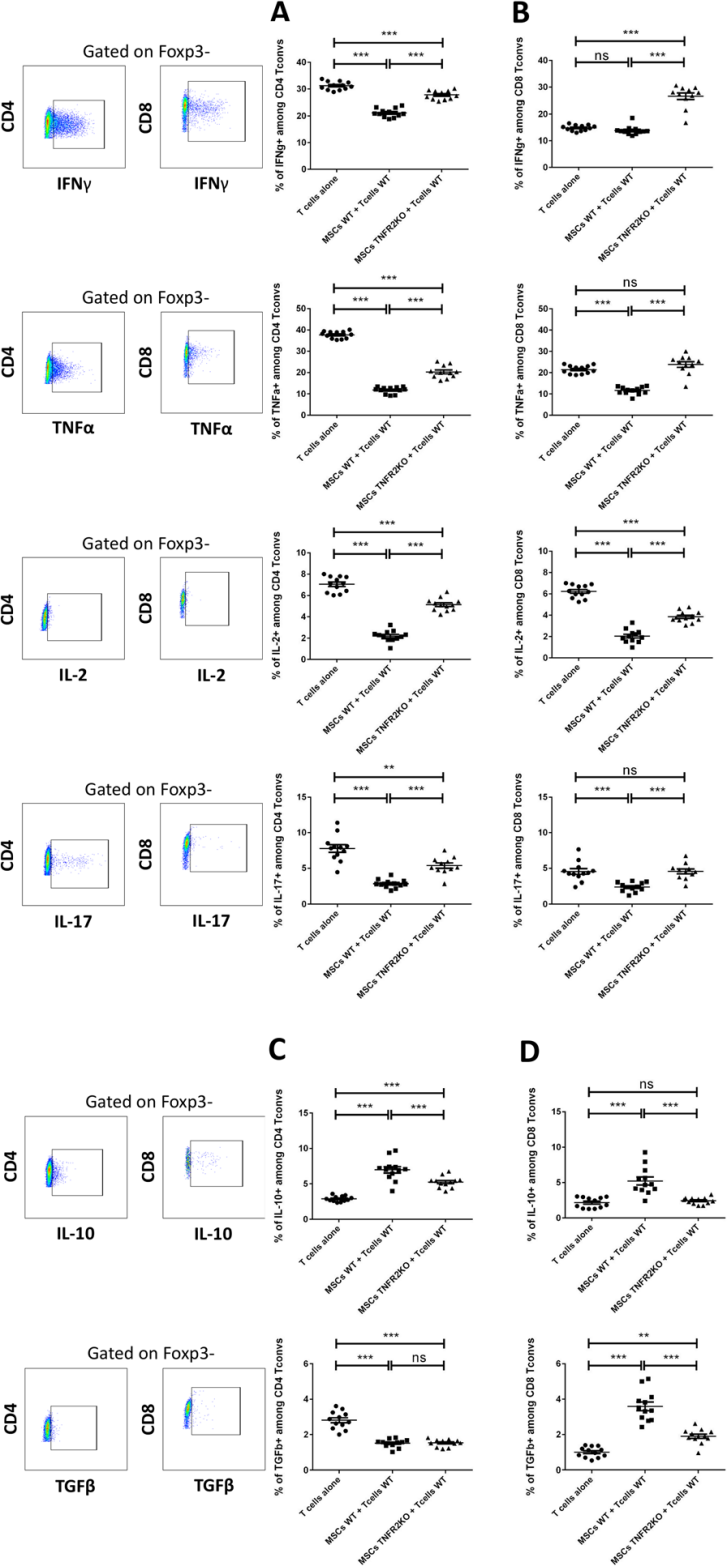
MSCs can modulate T cell capacity to produce pro or anti-inflammatory cytokines via the TNFα/TNFR2 signaling pathway. Effector T cells were co-cultured with MSCs WT or TNFR2 KO in a fixed 1/5 ratio. After 3 days, T cells were collected, activated with PMA/ionomycin, and then blocked with GolgiPlug (a protein transport inhibitor). Intracellular pro-inflammatory cytokine (**a**, **b**) and anti-inflammatory cytokine (**c**, **d**) production was determined in both CD4^+^ and CD8^+^ T cells. Cells were first gated on CD4^+^Foxp3^−^ or CD8^+^Foxp3^−^ T cells to precisely analyze the conventional T cell population. For each marker, the strategy of gating is indicated on the left (*y*-axis) and below (*x*-axis) the figure. The *x*-axis represents each cytokine, and the *y*-axis represents the CD4 or CD8 populations. Each dot represents a measured value (*n* = 12) collected from 2 different experiments. For each group of values, horizontal lines represent the mean value and standard error of the mean. One-way ANOVA analysis was performed to generate *P* values. ns, non-significant; **P < .01, ***P < .001

After activation, T cells produce pro- or anti-inflammatory cytokines. We examined whether blocking the TNFα/TNFR2 signaling pathway in MSCs modifies T cell cytokine production. We focused on principle cytokines secreted by the four main subpopulations of T helper cells (Th1, Th2, Th17, and T reg) and cytotoxic T cells (Tc1, Tc2, Tc17, and T reg). WT and TNFR2 KO-MSCs were co-cultured with WT-CD3^+^CD25^−^T cells. After 3 days, T cells were collected and analyzed for their cytokine production capacity. At first, we investigated the capacity of T cells to produce different pro-inflammatory cytokines. Interestingly, we observed a significant reduction in CD4^+^T convs producing IFNγ, TNFα, IL-2, and IL-17 when co-cultured with WT or TNFR2 KO-MSCs, compared to control T cells. This decrease was more significant for WT-MSCs compared to TNFR2 KO-MSCs (Fig. 5a). In CD8^+^T convs, we did not observe a significant decrease in IFNγ-producing T cells while co-cultured with WT-MSCs; however, there was a notable increase in IFNγ-producing CD8^+^T cells while co-cultured with TNFR2 KO-MSCs (Fig. 5b). Regarding TNFα and IL-17, a reduced production capacity was noticed merely by CD8^+^T convs co-cultured with WT-MSCs and not TNFR2 KO (Fig. 5b). Co-culture of CD8^+^T convs with WT or TNFR2 KO-MSCs led to a significant decrease in IL-2 production that was stronger with WT cells (Fig. 5b).

We then investigated whether co-culturing T convs with MSCs alters T cell anti-inflammatory cytokine production. We measured the production of IL-10 and TGFβ by T cells in the presence of MSCs. There was an increase in IL-10-producing cells in CD4^+^T cells co-cultured with WT or TNFR2 KO-MSCs, which was significantly higher with WT-MSCs (Fig. 5c). We found no increase in TGFβ-producing CD4^+^T cells co-cultured with either of MSCs (Fig. 5c). Additionally, we observed a significant increase in IL-10-producing CD8^+^T cells merely while co-cultured with WT-MSCs. Finaly, we noticed an increased TGFβ-producing CD8^+^T cells co-cultured with either of MSCs but remarkably higher for the WT-MSCs group (Fig. 5d).

### Expression of TNFR2 by MSCs is correlated to their higher Foxp3^+^T reg induction capacity

**Fig. 6:**
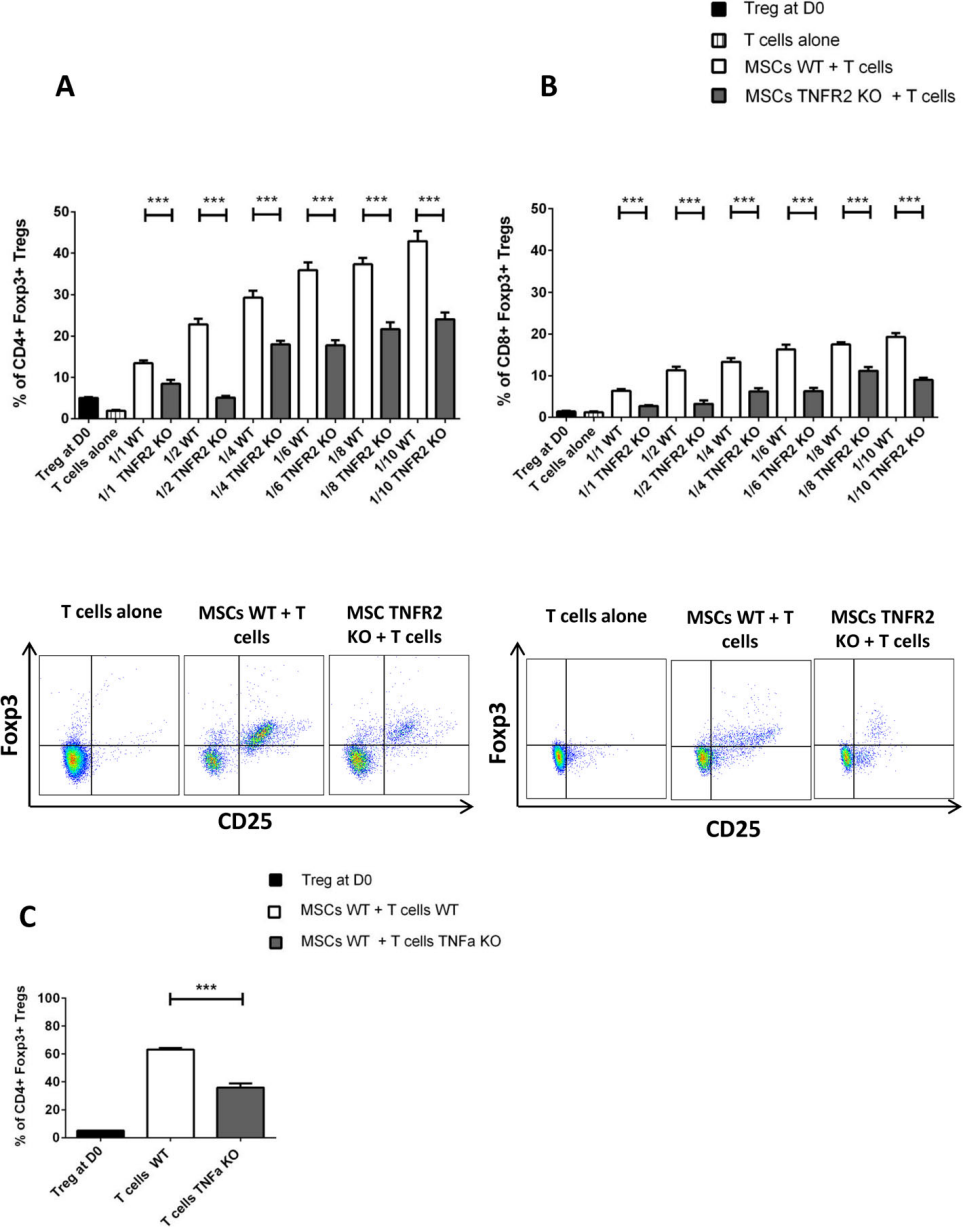
Expression of TNFR2 by MSCs is directly correlated to their higher Foxp3 T reg induction capacity. Activated CD4^+^ or CD8^+^ effector T cells were co-cultured with MSCs WT or TNFR2 KO in different ratios (*n* = 9). After 3 days, the expression of Foxp3 among CD4^+^ T cells (**a**) and CD8^+^ T cells (**b**) was measured by flow cytometry. Cells were gated on CD4^+^CD25^+^Foxp3^+^or CD8^+^CD25^+^Foxp3^+^ T cells. The gating strategy is indicated on the left and under the figures. The first black bar represents the percentage of Foxp3-expressing cells among total effector T cells used in co-cultures at day 0 (*n* = 9) while the second bar represents the percentage of Foxp3-expressing T cells alone after 3 days of culture (*n* = 9). (**c**) CD4^+^ T cells collected from the spleen of WT or TNFα deficient mice were co-cultured with MSCs WT. Cells were gated on CD4^+^CD25^+^Foxp3^+^. Data are represented as the mean value ± SEM collected from 3 different experiments. One-way ANOVA or unpaired Student *t* test analysis was performed to generate *P* values; ***P < .001. D0, day 0

We and others have clearly shown that one of the main immunoregulatory mechanisms of MSCs is the induction of T regs (iTregs) from T convs. Here, we focused on the role of the TNFα/TNFR2 signaling pathway in this process. To investigate the ability of MSCs to convert CD3^+^CD25^−^T convs to Foxp3^+^T regs, MSCs were co-cultured with anti-CD3/CD28-activated CD3^+^CD25^−^T cells at 6 different ratios. CD25^+^T cells were depleted to eliminate the unspecific expansion of natural T regs. After 3 days of co-culture, T cells were collected and analyzed for Foxp3 expression. In all conditions, both CD4^+^ (Fig. 6a) and CD8^+^T cells (Fig. 6b) in co-culture with WT or TNFR2 KO-MSCs expressed more Foxp3 than in the control T cells alone in a dose-dependent manner. In the CD4^+^ population, 1.89% of the T cells expressed Foxp3 at day 0, i.e., immediately after the elimination of CD25^+^ subpopulation (T cells alone). This value significantly increased after co-culturing with WT or TNFR2 KO-MSCs, reaching to 13.43% and 8.45%, respectively, in 1/1 ratio and kept rising until 42.93% and 24.07%, respectively, in 1/10 ratio (Fig. 6a). In the CD8^+^ population, 1.22% of the T cells expressed Foxp3 at day 0, after the elimination of CD25^+^ subpopulation. This value significantly increased after co-culturing with WT or TNFR2 KO-MSCs, reaching to 6.38% and 2.66%, respectively, in 1/1 ratio and kept rising until 19.30% and 9%, respectively, in 1/10 ratio (Fig. 6b).

To reinforce our results, we disrupted this signaling pathway via blocking the TNFα production by T cells. In this context, MSCs express TNFR2 but receive no signals from T cells. WT-MSCs were co-cultured with anti-CD3/CD28-activated CD3^+^CD25^−^T cells harvested from WT or TNFα KO mice in a fixed ratio (1/10 MSCs/T cells, i.e., the most efficient ratio to induce Foxp3 expression) for 3 days. As expected, the percentage of Foxp3^+^T regs among CD4^+^T convs was significantly less in TNFα KO-T cells compared to WT T cells (35.86% and 63.2%, respectively) (Fig. 6c). Thus, two different approaches confirm the critical role of the TNFα/TNFR2 signaling pathway in T reg induction by MSCs.

### Expression of TNFR2 on MSCs leads to induction of T regs with a more active phenotype

**Fig. 7:**
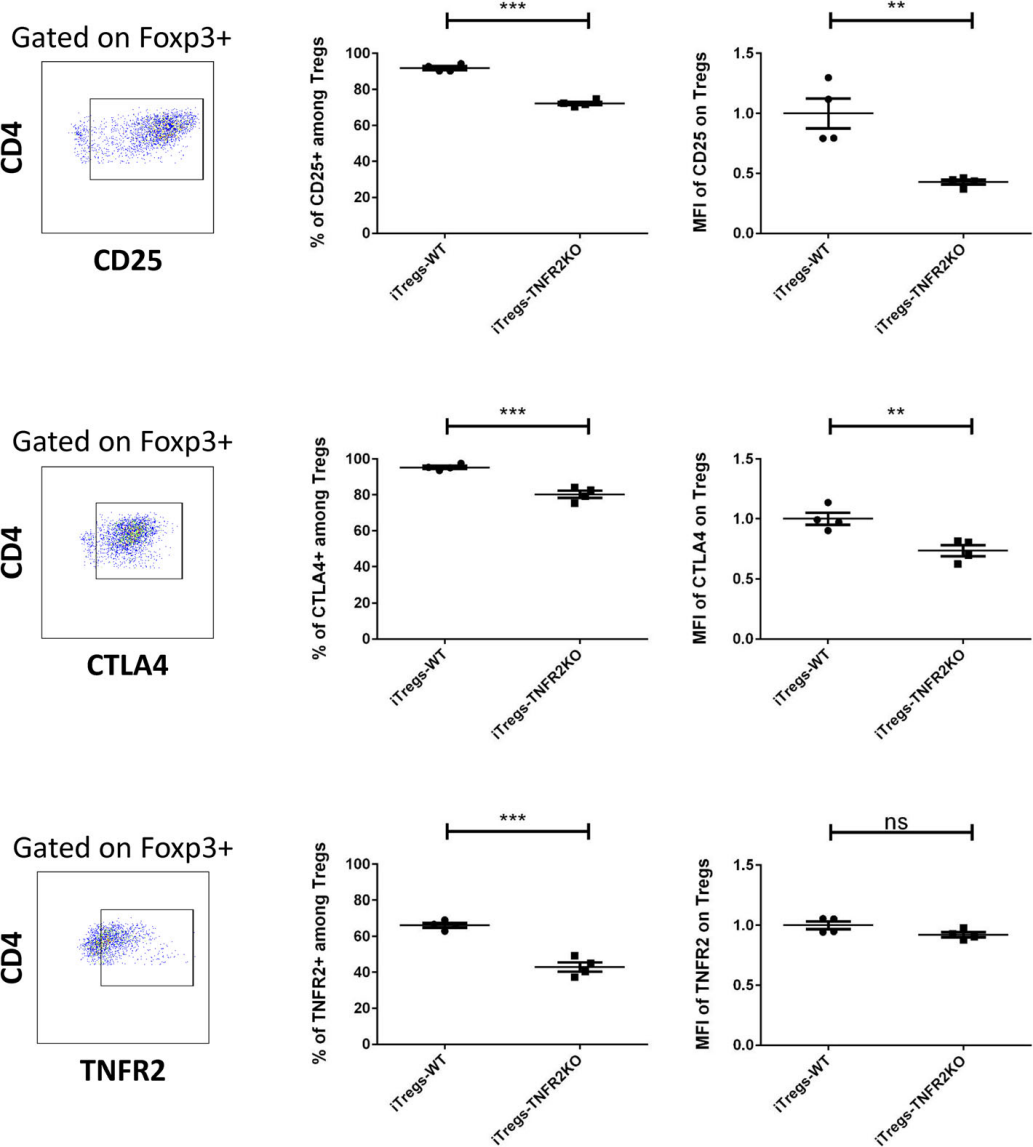
The expression of TNFR2 on MSCs is leading to the induction of T regs with a more activated phenotype. The activation status of the T regs that were induced after the co-culture of CD4^+^ conventional T cells with MSCs WT or TNFR2 KO was evaluated. Cells were gated on CD4^+^Foxp3^+^T cells. For each marker, the strategy of gating is indicated on the left and under the figure. Each dot represents a measured value (*n* = 4) collected from 2 different experiments. For each group of values, horizontal lines represent the mean value and standard error of the mean. Unpaired Student *t* test analysis was performed to generate *P* values. ns, non-significant; **P < .01, ***P < .001. iTregs, induced T reg cells

To characterize the activation profile of CD4^+^Foxp3^+^iTregs, we quantified different activation markers, such as CD25, CTLA4, and TNFR2. The percentage of CD25^+^ cells and the CD25 expression level on CD4^+^Foxp3^+^iTregs was significantly higher after co-culture with WT-MSCs than with TNFR2 KO-MSCs (Fig. 7). The same results were observed for the CTLA4 expression (Fig. 7). We and others have shown that one of the most important regulators of T reg activity is TNFR2 which is directly related to their activation and immunosuppressive function [38, 52]. We found that the percentage of TNFR2^+^iTregs from co-culture with WT-MSCs was significantly higher than TNFR2 KO-MSCs (Fig. 7). No significant difference was observed for MFI of TNFR2 in different conditions. Thus, TNFR2^+^MSCs were able to induce more active Foxp3^+^T regs compared to TNFR2^−^ MSCs.

## Discussion

Since MSCs display wound healing [53], immunomodulatory, and anti-inflammatory effects [25–27], they are ideal choices for cell therapy applications. First clinical trials were performed with autologous MSCs, but those treatments were patient-specific, inefficient, and expensive [54]. Then, converging evidences showed that allogenic MSCs have comparable efficacy, without immune rejection issues [55]. This established interesting perspectives for broader administration of MSCs in clinics using banks of allogenic MSCs from different tissue origins. Therefore, it is crucial to understand the mechanisms behind MSC immunoregulatory activity.

Here, we performed co-cultures of MSCs (WT and TNFR2 KO) and T cells (WT and TNFα KO) to investigate the effects of the TNFα/TNFR2 axis on MSC-T cell interaction. We have previously assessed and reported the viability of MSCs and T cells upon co-culturing in different conditions. The viability of cells was between 77 and 98% depending on the co-culture condition [25–27].

Co-culture of activated CD4^+^Foxp3^−^ and CD8^+^Foxp3^−^T cells with MSCs remarkably reduced their proliferation in a dose-dependent manner. Interestingly, this immunosuppressive effect was significantly decreased when TNFR2 KO-MSCs were used. Our data point that the TNFα/TNFR2 axis is an important but not the only regulator of MSC immunosuppressive function since TNFR2 KO-MSCs were also immunosuppressive but less efficiently. We then measured the ability of MSCs to modify T conv activation profile by quantifying the expression of CD25, GITR, ICOS, and TNFR2 markers. While both MSCs were able to down-modulate CD4^+^Foxp3^−^ and CD8^+^Foxp3^−^T cell activation, this immunomodulatory effect was stronger with WT than TNFR2 KO-MSCs. Thus, we report a direct correlation between the TNFR2 expression and the MSC immunomodulatory effect. Among different T cell activation markers, we targeted two TNFα receptor superfamily members, GITR and TNFR2, and demonstrated a more significant decrease in their expression while T cells were co-cultured with WT-MSCs. This reflects a complex modulation of TNFα signaling in T cells in the presence of TNFR2^+^MSCs. Accordingly, other studies showed when TNFR2 is decreased on T cells, they will be more efficiently suppressed by T regs [50] and here by MSCs. Furthermore, this downregulation was accompanied by a modification in T cell cytokine production. WT-MSCs decreased T cell pro-inflammatory cytokines like IFNγ, TNFα, IL-2, and IL-17 and increased the anti-inflammatory cytokines like IL-10 and TGFβ. This effect was less or even totally hindered with TNFR2 KO-MSCs, pointing out the critical role of TNFR2 expression by MSCs. This is in agreement with studies reporting, upon TNFα activation, MSCs produce a higher level of immunosuppressive molecules such PGE2, TSG6, IL-8, CXCL5, CXCL6, and other growth factors like HGF, IGF1, and VEGF [9, 56, 57].

Thereafter, we quantified the involvement of the TNFα/TNFR2 signaling pathway in the ability of MSCs to convert T convs to CD4^+^Foxp3^+^ and CD8^+^Foxp3^+^T regs. Expectedly, this effect was more preeminent with TNFR2^+^MSCs than with TNFR2 KO-MSCs. These results are encouraging, since CD8^+^T regs are naturally rare population compared to their CD4^+^ counterparts. However, after co-culturing with TNFR2^+^MSCs, 20% of CD8^+^T cells expressed Foxp3 which is double compared to TNFR2 KO-MSCs. To reinforce our results, we hampered the TNFα/TNFR2 axis by co-culturing WT-MSCs and TNFα KO-T convs that are incapable of TNFα production. Again, in the absence of TNFα, the ability of T convs to differentiate into T regs was significantly decreased. Accordingly, it was demonstrated that pre-treatment of MSCs with TNFα is crucial for the polarization of macrophages towards more anti-inflammatory M2 and less pro-inflammatory M1 subpopulation [57] and increased T reg repertory [58].

As demonstrated in the results, even TNFR2 KO-MSCs show some level of immunoregulatory features; however, these effects are significantly less than WT-MSCs. We believe that the presence of TNFR2 guarantees higher survival and less apoptosis rate. The expression of TNFR2 has been already correlated to increased IL-10 and TGFβ production [40, 59]. As we have also shown, WT-MSCs are able to produce more of those anti-inflammatory cytokines, exerting more immunosuppressive effect. The absences of TNFR2 can negatively affect signaling through TNFR1 not only by decreasing functional cells but also by increasing the pro-inflammatory profile of surviving cells. It has been demonstrated that TNFR1 signaling promotes inflammation due to increased production of pro-inflammatory factors including TNF, IL-1β, and IL-6 [60]. However, observing a certain level of immunoregulation by TNFR2 KO-MSCs reveals some form of crosstalk between the two receptors. Due to the absence of the death domain (DD) in the TNFR2 signaling pathway; TNFR1 and TNFR2 have two distinct functionalities. Nevertheless, both receptors (predominantly TNFR2) can activate the NF-κB signaling pathway via the involvement of TNF receptor-associated factors (TRAFs) [61]. It has been demonstrated that TNFR family co-stimulation increases regulatory T cell activation and function via NF-κB [62]. Moreover, it was shown that the NF-κB RelA subunit (p65) transcription factor is critical for T reg activation and stability [63]. This could explain why TNFR2 KO-MSCs maintain a certain level of immunoregulation and are still able to induce T regs.

TNFR2 is expressed by most of the known immunosuppressive cells. Our results are the first evidence proving TNFR2 as an active immune checkpoint for MSCs, enabling regulation of their immunological features. It was shown that TNFR2^+^T regs are the most homogeneous population with the highest immunosuppressive function [64, 65]. MSCs are very heterogeneous stem cells, and this effects their functionality. It will be interesting to sort TNFR2^hi^MSCs to have the purest and the most immunosuppressive population.

In case of cancer, MSCs help tumor cells to evade immune attack, while promoting tumor angiogenesis [66]. They participate in many crucial steps from invasion and metastasis, including stimulating the epithelial-mesenchymal transition and induction of stem-like properties that allow cancer stem cells to increase their survivability [67, 68]. Using anti-TNFR2 treatment could be an efficient way to simultaneously hamper immunosuppression by T regs [38, 69] and other immunosuppressive cells present in the tumor microenvironment [70], to directly eliminate TNFR2 expressing tumor cell [71, 72], to interrupt angiogenesis [73], and to decrease MSC survival and function.

## Conclusions

Our results reveal another mechanism that MSCs use to regulate immune response. Using different in vitro experimental approaches, we have demonstrated for the first time that the TNFα/TNFR2 signaling pathway is in control of the immunomodulatory properties of MSCs. Signaling through this immune checkpoint axis can modulate MSC ability to suppress T cell proliferation, activation, cytokine production, and their capacity to induce active T regs.

This work brings us one step closer to this conclusion that targeting TNFR2 using its proper antagonist is an effective way to direct immunoregulatory responses. Not only will it efficiently control immunosuppression by MSCs and other TNFR2^+^ immunosuppressive cells but also angiogenesis and cell survival, highlighting its important therapeutic potential in cases like cancer treatment. Inversely, boosting through TNFR2 via administration of its agonist could potentially elevate MSC immunoregulatory function favoring the cases such as transplantations in which increased anti-inflammatory responses are especially required.

## Data Availability

The datasets used and/or analyzed during the current study are available from the corresponding authors on reasonable request.

## Abbreviations

B reg: Regulatory B cell
BM-MSCs: Bone marrow-derived mesenchymal stem cells
CD: Cluster of differentiation
CFSE: Carboxyfluorescein succinimidyl ester
DD: Death domain
EPCs: Endothelial progenitor cells
HGF: Hepatocyte growth factor
ICOS: Inducible co-stimulatory molecule
IFNγ: Interferon gamma
IGF-1: Insulin-like growth factor 1
IL: Interleukin
iTreg: Induced regulatory T cell
mAB: Monoclonal antibody
MACS: Magnetic-activated cell sorting
MDCSs: Myeloid-derived suppressive cells
MFI: Mean fluorescence intensity
MSCs: Mesenchymal stem cells
P: Passage
Sca1: Stem cell antigen-1
sTNFR2: Soluble tumor necrosis factor receptor 2
T conv: Conventional T cell
T eff: Effector T cell
T reg: Regulatory T cell
Tc: Cytotoxic T cells
Th: T helper cells
TNFR1: Tumor necrosis factor receptor 1
TNFR2 KO: Tumor necrosis factor receptor 2 knocked out
TNFR2: Tumor necrosis factor receptor 2
TNFα: Tumor necrosis factor alpha
TRAF: TNF receptor-associated factor
VEGF: Vascular endothelial growth factor
